# Samul-tang ameliorates oocyte damage due to cyclophosphamide-induced chronic ovarian dysfunction in mice

**DOI:** 10.1038/s41598-020-79013-7

**Published:** 2020-12-14

**Authors:** Jihyun Kim, Sooseong You

**Affiliations:** Clinical Medicine Division, Korea Institute of Oriental Medicine, 1672 Yuseongdae-ro, Yuseong-gu, Daejeon, 34054 Republic of Korea

**Keywords:** Developmental biology, Genetics, Molecular medicine

## Abstract

Samul-tang (SM), a traditional herbal medicine, has been used to treat menstrual irregularities and infertility in women. However, the cellular and molecular mechanisms underlying the effects of SM remain elusive. We investigated the potential protective effect of SM against chronic ovarian dysfunction and used bioinformatics analysis to identify its underlying mechanism in a mouse model of cyclophosphamide (CP)-induced diminished ovarian reserve. Female C57BL/6 mice were intraperitoneally injected with CP three times a week, followed by oral administration of distilled water (CP group) or SM (CP + SM group) for 4 weeks. Four weeks later, the effect of SM was assessed by ovarian tissue histological analysis, steroid hormone measurement, oocyte quality, and mRNA and microRNA microarray analysis in the ovaries. Although SM administration did not prevent CP-induced follicle loss in mice, the quality of oocytes was better in CP + SM mice than in CP mice. Gene expression analysis revealed that the expression of fertilisation- and ovarian follicle development-related genes was altered by CP treatment but normalized after SM administration. Further bioinformatics analysis showed possible interactions between differentially expressed mRNAs and microRNAs. Therefore, we demonstrated the protective effects of SM on ovarian function and oocyte maturation against CP-induced damage via multiple epigenetic mechanisms.

## Introduction

Extensive research is currently being conducted to develop alternative protective options against various devastating impacts of chemotherapy, surgery, and endocrine-disrupting chemicals on reproductive health^1^. In particular, herbal formulas have been proven to prevent and treat many diseases by promoting recovery and ameliorating impaired functions^2^. Samul-tang (SM), consisting of equal proportions of four herbs, *Paeonia lactiflora, Ligusticum striatum, Rehmannia glutinosa,* and *Angelica gigas*, is a well-known mixed herbal medicine. In in vivo and in vitro human and rodent models, SM has been reported to exert biological protective effects against haematological disorders^3^, oxidative stress^4^, apoptotic cell death^5^, and inflammation^6^. Furthermore, SM is commonly prescribed to women with gynaecological disorders, such as irregular menstruation and postmenopausal syndrome, which often occur in individuals with diminished ovarian reserve (DOR)^7^.

DOR is defined as a decrease in the quantity and quality of mature oocytes, leading to infertility^8^. Women with DOR often have low blood flow to the ovaries, which has a negative consequence for competence^9^. The beneficial effect of SM on blood circulation has been reported^10^, even though the molecular mechanisms of action remain elusive. The aetiology of DOR includes previous ovarian surgery, exposure to toxic chemicals, chemotherapy, and radiation^11^. Repeated monthly chemotherapy administration results in folliculogenesis failure and severely impairs ovarian function^12^. In particular, cyclophosphamide (CP) exposure is associated with a long-term risk of premature ovarian failure and early menopause^13,14^. Further, CP exposure induces oxidative stress in spermatogenetic cells, granulosa cells and oocytes, thereby inducing organ toxicity^15^. Herbal medicine was found to attenuate CP-induced organ toxicity via anti-oxidative and anti-apoptosis pathways^16^.

With the growing numbers of young female cancer survivors and the aim of fertility preservation, the maintenance of reproductive potential after cancer therapy has become a paramount concern. Although herbal medicines are traditionally known to improve fertility-related parameters, scientific evidence on their use in adjuvant therapy is limited. Therefore, in this study, we investigated the potential protective effect of SM and its underlying molecular mechanism in mice with a CP-induced chronic impairment of ovarian function.

## Results

### SM reduced CP-induced impairment of hormonal regulation

**Figure 1:**
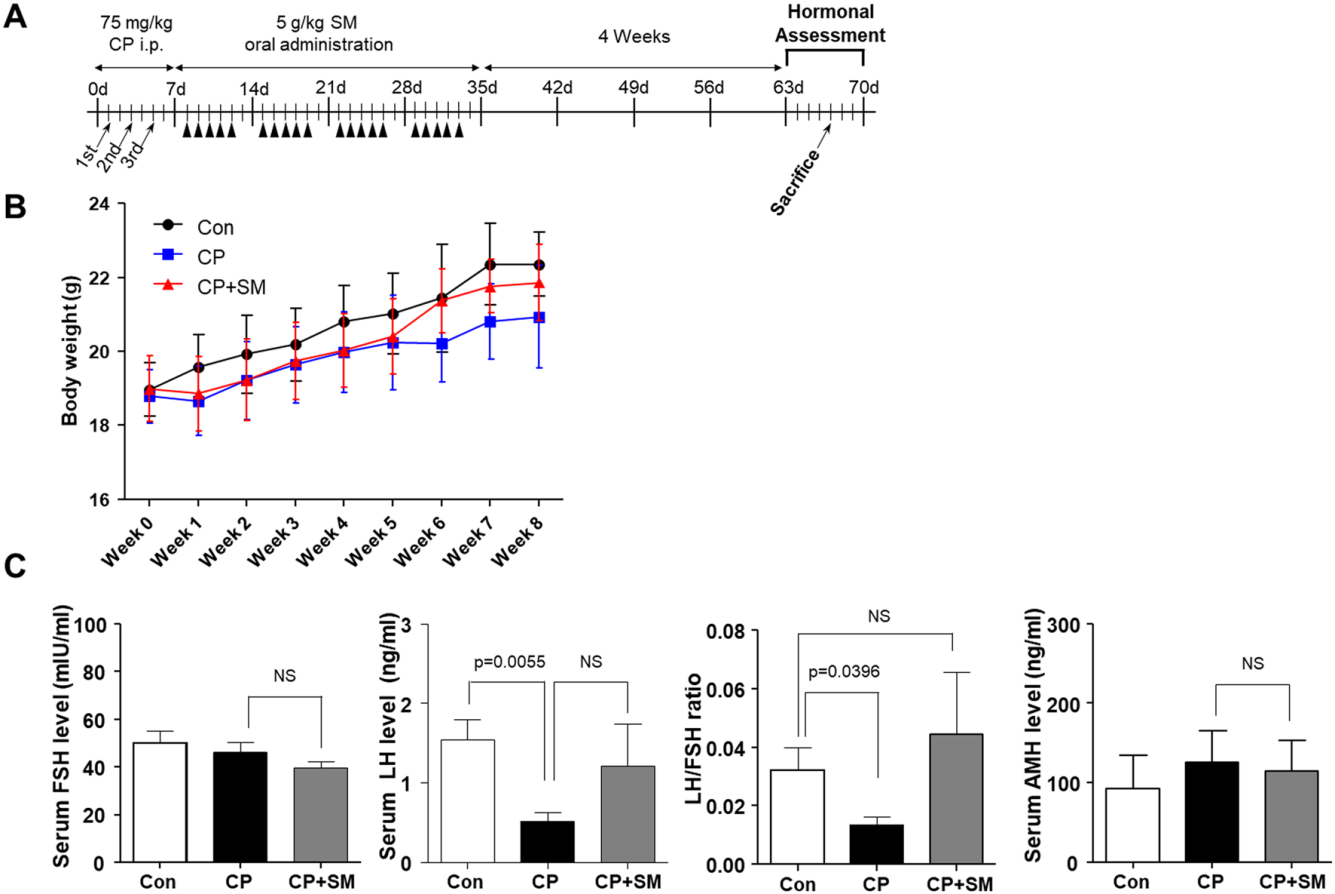
Body weight changes and serum hormone levels in mice at 4 weeks after Samul-tang (SM) administration. (**A**) Mice were intraperitoneally injected with saline (n = 4, Con group) or cyclophosphamide (CP) on days 1, 3 and 5 in the first week; mice injected with CP were then orally administered distilled water (n = 5, CP group) or 5 g/kg SM (n = 5, CP + SM group) five times a week for 4 weeks. Four weeks after SM administration, the mice were weighed, and hormonal assessment was performed. (**B**) Body weight changes. (**C**) Serum levels of follicle-stimulating hormone (FSH), luteinising hormone (LH), and anti-Müllerian hormone (AMH), and the LH/FSH ratio. Statistical analysis was performed using the Student’s *t*-test. Arrows and arrowheads indicate the days of CP injection and SM administration, respectively. *NS* not significant.

The mice were monitored weekly throughout the study (Fig. 1A). The body weights of the CP-treated mice rapidly decreased during the week of CP administration and recovered only slightly after the cessation of CP exposure (Fig. 1B). SM administration resulted in a progressive, although not statistically significant, increase in body weight.

We next measured the serum follicle-stimulating hormone (FSH), luteinising hormone (LH), and anti-Müllerian hormone (AMH) levels in the Con, CP, and CP + SM mice. The levels of FSH and LH did not differ between CP and CP + SM mice. The CP mice had low LH levels, resulting in a decreased LH/FSH ratio (Fig. 1C). However, SM administration restored the LH/FSH ratio to a value comparable with that in the control (Con) mice. The serum AMH levels in the CP + SM mice were not markedly different from those in the CP and Con mice.

## SM did not prevent CP-induced follicle loss

**Figure 2:**
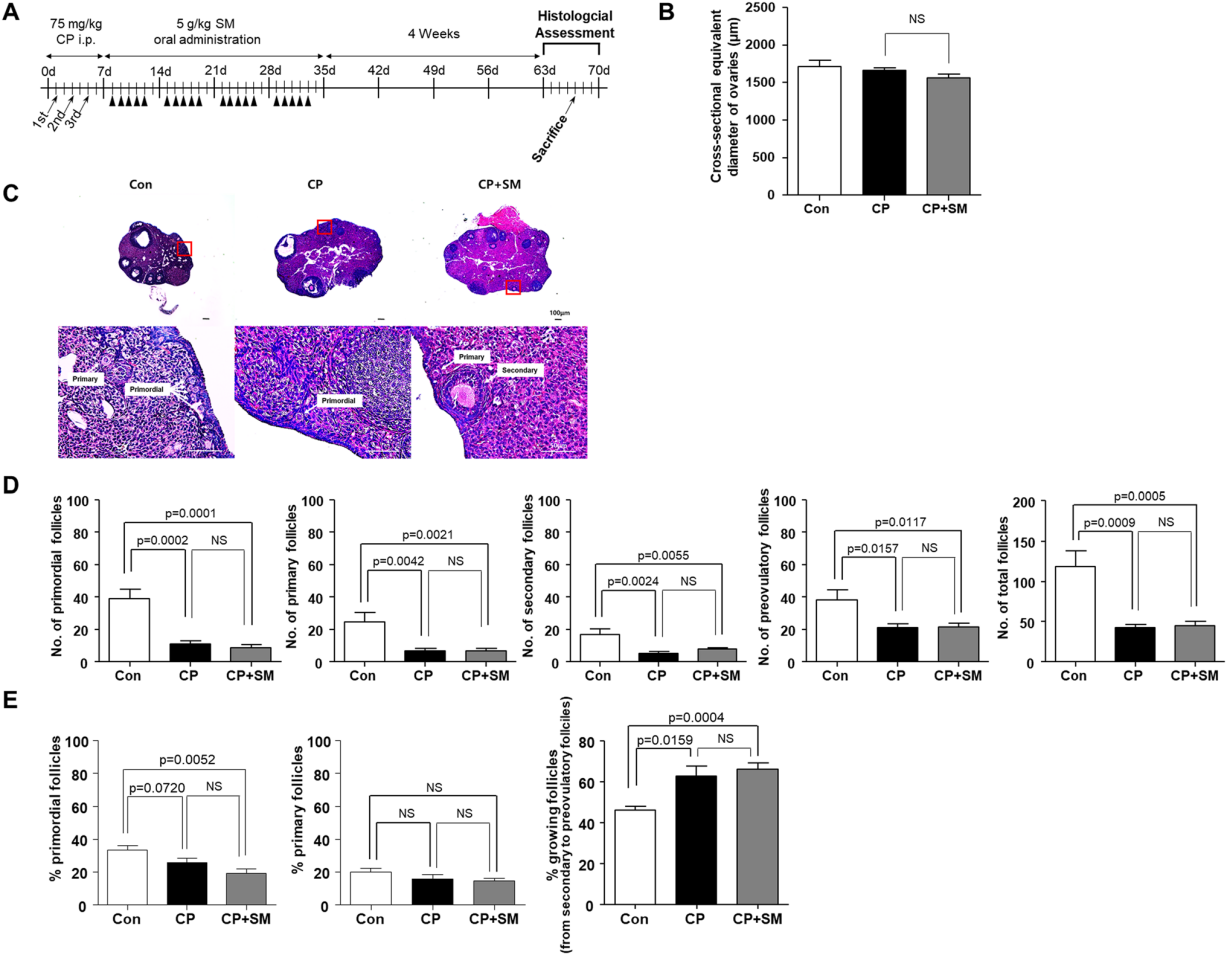
Histological analysis of ovarian follicles in mice at 4 weeks after Samul-tang (SM) administration. (**A**) Mice were intraperitoneally injected with saline (n = 4, Con group) or cyclophosphamide (CP) on days 1, 3, and 5 in the first week; mice injected with CP were then orally administered distilled water (n = 4, CP group) or 5 g/kg SM (n = 5, CP + SM group) five times a week for 4 weeks. Four weeks after SM administration, ovaries from all three groups were assessed histologically. (**B**) Cross-sectional equivalent diameter of the ovaries. (**C**) Representative histological images of ovaries from mice in the three experimental groups. (**D**) Numbers of ovarian follicles in different stages and the total number of ovarian follicles; and (**E**) proportions of primordial, primary, and growing (secondary and preovulatory) follicles in the Con, CP, and CP + SM mice. Statistical analysis was performed using the Student’s *t*-test. Arrows and arrowheads indicate the days of CP injection and SM administration, respectively. *NS* not significant.

To investigate whether SM exerts a protective effect on ovarian follicle growth under conditions of CP exposure, we performed a histological analysis of the ovaries excised from the mice at 4 weeks after SM administration (Fig. 2A). The cross-sectional equivalent diameters of the ovaries were similar among the three mouse groups (Fig. 2B). Histological changes induced by CP exposure were observed in the ovarian tissues (Fig. 2C). Compared to the Con mice, the CP and CP + SM mice showed lower total numbers of follicles at different stages (Fig. 2D). Furthermore, the latter two groups showed significantly higher proportions of growing (secondary and preovulatory) follicles (*P* < 0.05; Fig. 2E). The follicular growth was not fount to differ between the CP and CP + SM mice. Thus, SM administration did not prevent the reduction of the primordial follicle pool and the CP-induced burnout phenomenon^17^.

### SM ameliorated CP-induced impairment of oocyte quality

**Figure 3:**
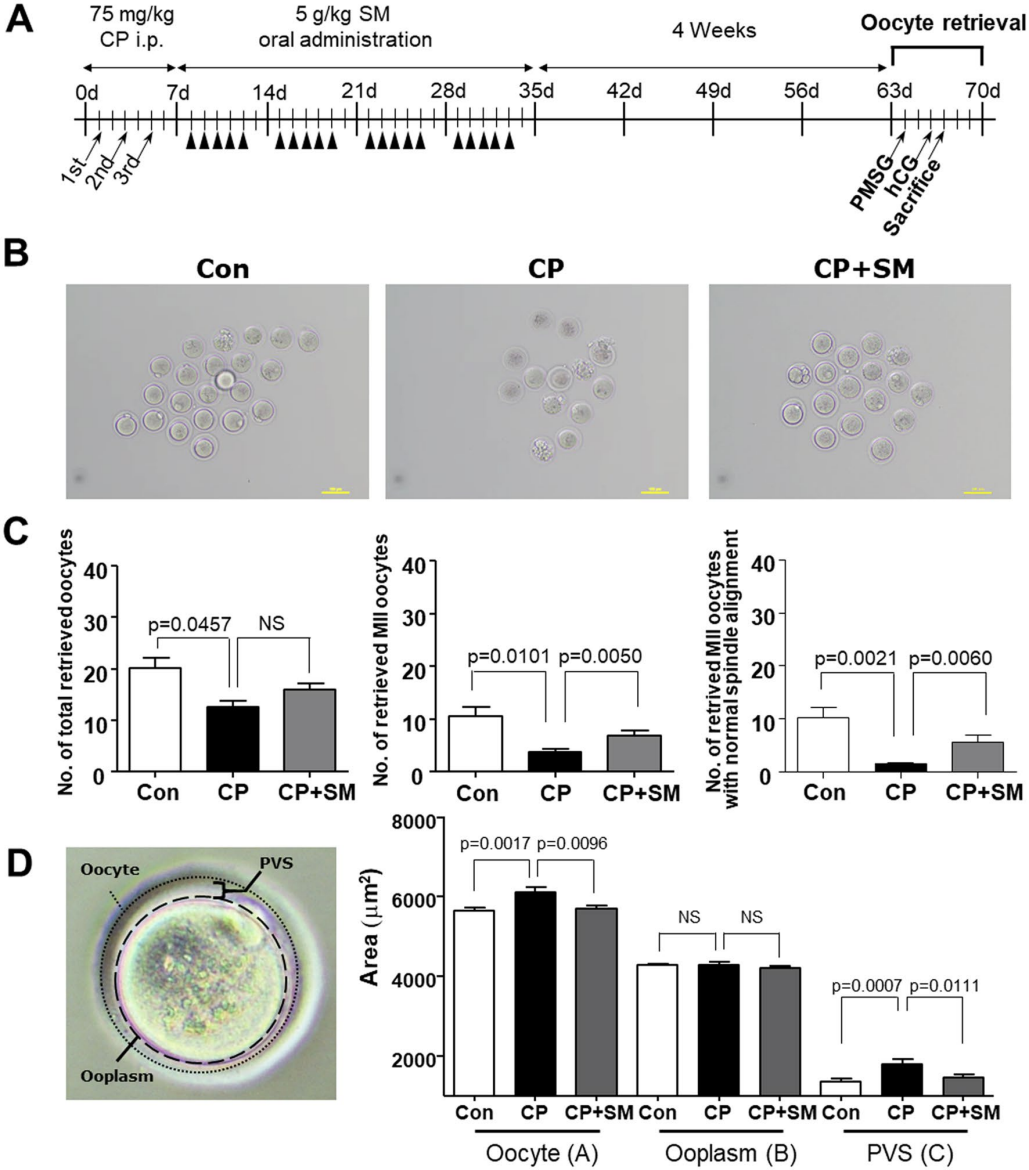
Quality and quantity of mouse oocytes retrieved at 4 weeks after Samul-tang (SM) administration. (**A**) Mice were injected with saline (n = 5, Con group) or cyclophosphamide (CP) on days 1, 3, and 5 in the first week; mice injected with CP were then orally administered distilled water (n = 5, CP group) or 5 g/kg SM (n = 5, CP + SM group) five times a week for 4 weeks. Four weeks after SM administration, the mice were superovulated via intraperitoneal injections of 5 IU of pregnant mare serum gonadotropin (PMSG) and 5 IU of human chorionic gonadotropin (hCG) at 48 h after PMSG injection. (**B**) Oocytes retrieved at 18 h after hCG injection from the Con, CP, and CP + SM mice. (**C**) Numbers of oocytes, MII oocytes, and MII oocytes with normal chromosomal and spindle alignment, retrieved from the three mouse groups. (**D**) Areas of oocytes, the ooplasm, and perivitelline space (PVS) in the three mouse groups. Statistical analysis was performed using the Student’s *t*-test. Scale bars indicate 100 µm. Arrows and arrowheads indicate the days of CP injection and SM administration, respectively. *NS* not significant.

To investigate whether SM exerts a protective effect on oogenesis under conditions of CP exposure, the mice were hormonally superovulated (Fig. 3A). Oocytes were collected from the oviducts at 18 h post-hCG administration, and their quantity and quality were assessed. Oocytes from CP mice showed ZP defect as loosely compacted and enlarged (Fig. 3B). The numbers of total oocytes and mature metaphase II (MII) oocytes retrieved from the CP mice were significantly lower than those retrieved from the Con mice (*P* < 0.05; Fig. 3C). The number of MII oocytes, with normal chromosomes and well-organised spindle alignments, was significantly higher in the CP + SM mice than in the CP mice displaying chromosomal abnormalities and spindle misalignments (Fig. 3C).

Moreover, CP treatment led to an increase in oocyte size and an enlargement of the perivitelline space (PVS). These effects were reversed upon SM administration so that the CP + SM mice had PVS and ooplasm areas similar to those in the Con mice (Fig. 3D). These results indicate that SM can protect the quality of superovulated oocytes even at 4 weeks after the completion of SM administration.

### SM reversed CP-induced changes in mRNA expression in mouse ovaries

**Figure 4:**
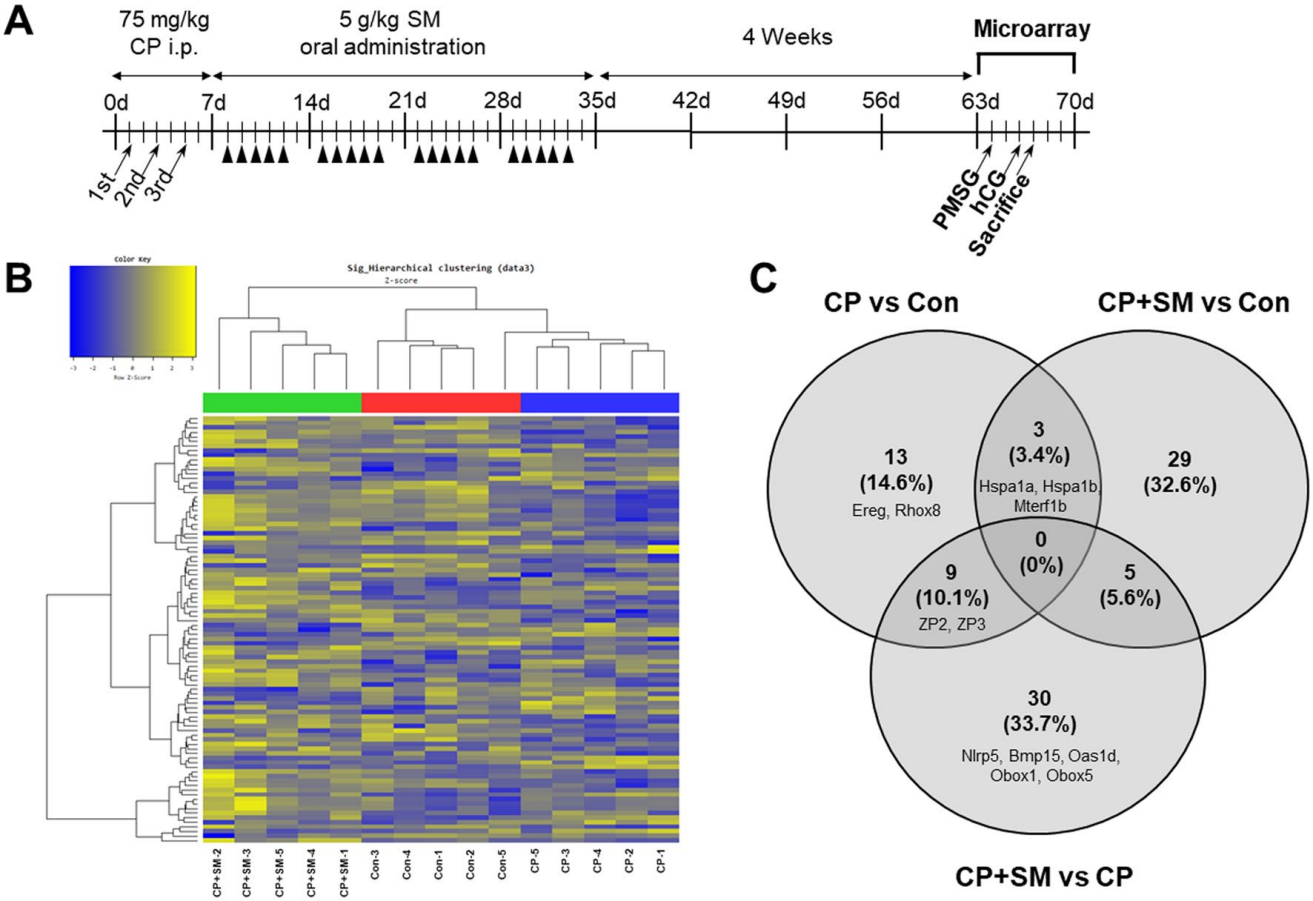
Hierarchical clustering and analysis of differentially expressed mRNAs. (**A**) Microarray was performed to compare the gene expression in ovulated ovaries of Con (n = 5), CP (n = 5), and CP + SM mice (n = 5). (**B**) Hierarchical clustering among the mRNA expression profiles. (**C**) Venn diagram showing 89 differentially expressed mRNAs in the three groups, with a fold-change > 1.5 and *P* < 0.05. *Con* saline-treated control mice; *CP* cyclophosphamide-treated mice; *CP + SM* CP-treated mice orally administered Samul-tang. Arrows and arrowheads indicate the day of CP injection and SM administration, respectively.

We next performed a microarray analysis to compare the mRNA expression patterns in the ovulated ovaries from the Con, CP, and CP + SM mice (Fig. 4A). Hierarchical clustering analysis showed marked differences among the three mouse groups (Fig. 4B), with 33,793 genes displaying significantly different expression levels in at least one comparison set.

Of the 33,793 genes identified, 89 showed significantly altered expression, with fold changes > 1.5 (*P* < 0.05). The numbers of differentially expressed genes (DEGs) between the three mouse group pairs are presented in a Venn diagram (Fig. 4C). Comparison of the Con and CP mice data revealed 13 DEGs, including the *Ereg* and *Rhox8* genes, which are involved in ovarian folliculogenesis and oocyte ovulation (Fig. 4C, Supplementary Fig. S1A). Compared to the Con mice, both CP and CP + SM mice showed significant increases in the expression of three genes, *Hspa1b*, *Hspa1a*, and *Mterf1b* (Fig. 4C, Supplementary Fig. S1B). Gene expression was validated by qPCR using fluorescent probe-based Taqman assays (Supplementary Fig. S1). We found that expression patterns of *Rhox8* and *Mterf1b* were different compared to microarray data. These genes are involved in the regulation of mitotic spindle assembly and protein refolding. These results, indicating CP-induced differential gene expression, might explain the presence of abnormal oocytes, with disarranged spindle alignment, resulting in fertilisation failure.

**Figure 5:**
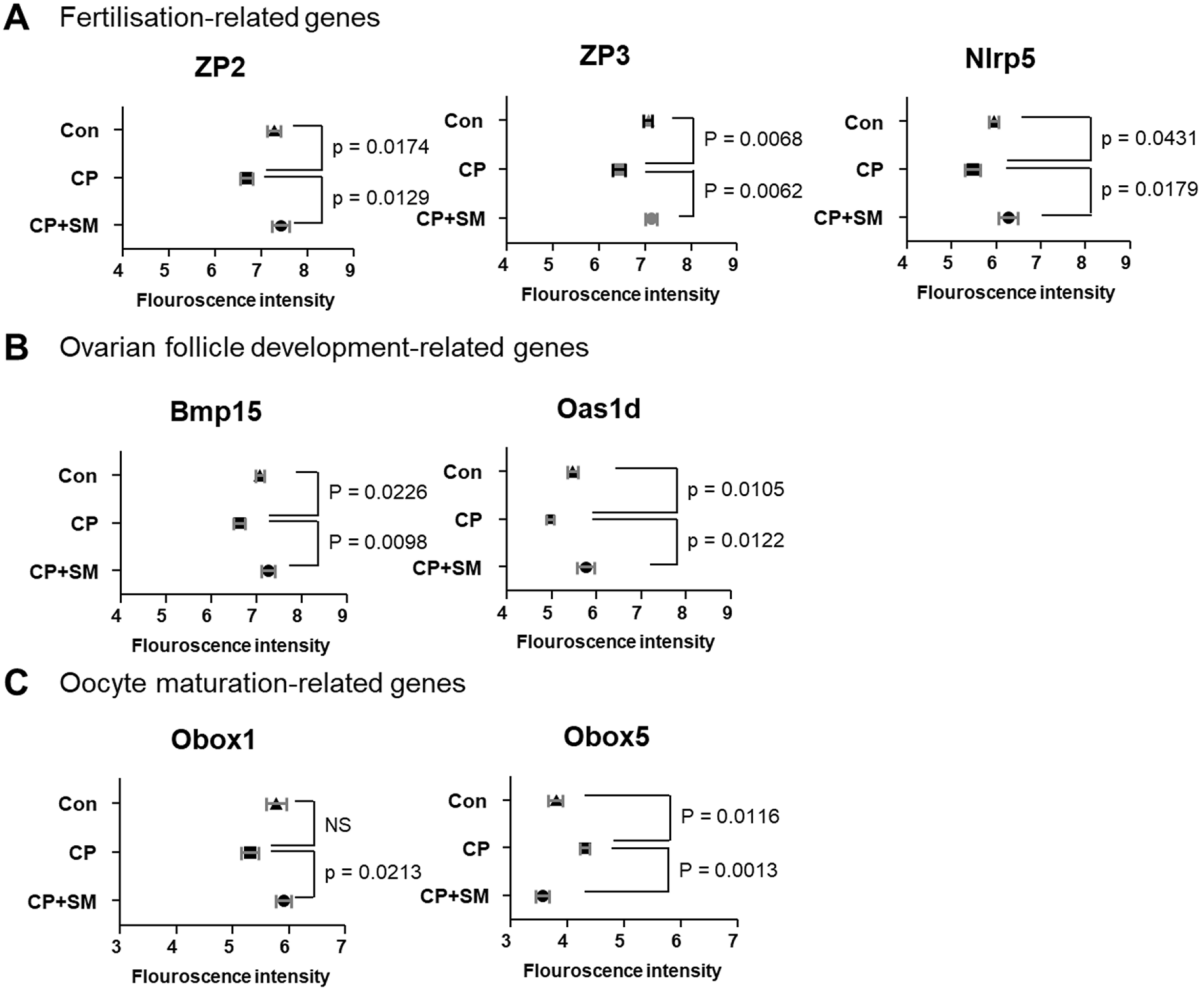
Differentially expressed mRNAs involved in ovarian function. Microarray fluorescence intensities indicate the expression levels of (**A**) *Zp2*, *Zp3*, and *Nlrp5* genes, involved in fertilisation, (**B**) *Bmp15* and *Oas1d* genes, involved in ovarian follicle development, and (**C**) *Obox1* and *Obox5* genes, involved in oocyte maturation, in ovaries of the Con, CP, and CP + SM mice. Statistical analysis was performed using the Student’s *t*-test. *Con* saline-treated control mice; *CP* cyclophosphamide-treated mice; *CP + SM* CP-treated mice orally administered Samul-tang.

**Figure 6:**
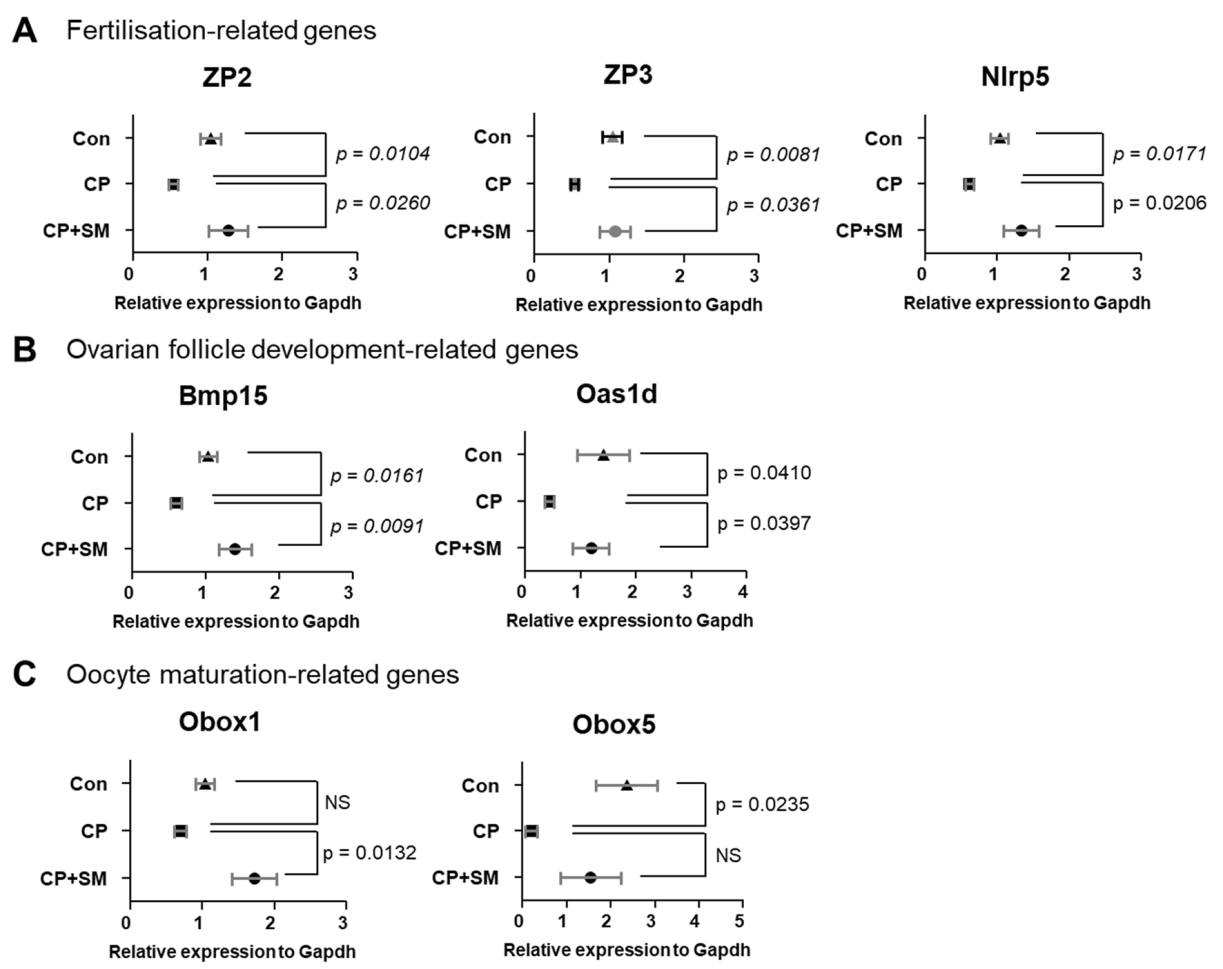
Validation of the expression of differentially expressed mRNAs involved in ovarian function. qPCR was performed to validate the expression of (**A**) *Zp2*, *Zp3*, and *Nlrp5* genes involved in fertilisation, (**B**) *Bmp15* and *Oas1d* genes, involved in ovarian follicle development, and(**C**) *Obox1* and *Obox5* genes, involved in oocyte maturation, in the ovaries of the Con, CP, and CP + SM mice. Statistical analysis was performed using the Student’s *t*-test. Con: saline-treated control mice; *CP* cyclophosphamide-treated mice; *CP + SM* CP-treated mice orally administered Samul-tang.

Compared to the CP mice, the CP + SM mice showed 44 significant DEGs, of which 31 (70.5%) were upregulated and 13 (29.5%) were downregulated (Supplementary Tables S1 and S2). Interestingly, nine genes overlapped between the CP vs Con and CP + SM vs CP pairs (Fig. 4C). Of these, the zona pellucida glycoprotein 2 and 3 genes (*Zp2* and *Zp3*), which play an important role in fertilisation, were downregulated in the CP mice, but their expression levels were restored by SM administration (Fig. 5A). Thirty DEGs which were differentially expressed only in CP + SM vs CP mice included NLR family pyrin domain containing 5 (*Nlrp5*), bone morphogenetic protein 15 (*Bmp15*), 2′-5′ oligoadenylate synthetase 1D (*Oas1d*), and oocyte-specific homeobox 1 and 5 (*Obox1* and *Obox5*) (Fig. 4C). These genes are known to be involved in fertilisation, folliculogenesis, and oocyte maturation (Fig. 5A–C). The expression of these genes was validated by qPCR with fluorescent probe-based Taqman assays (Fig. 6A–C). We found that the expression pattern of *Obox5* was different compared to microarray data. CP treatment significantly altered the expression of these five genes in the ovaries, whereas their expression levels in the ovaries of the CP + SM mice were comparable to those in the ovaries of the Con mice. These findings suggest that SM administration ameliorated the CP-induced impairment of ovarian function via genetic regulation. The five DEGs that overlapped between the CP + SM vs Con and CP + SM vs CP pairs were associated with immunoglobulin formation and immune responses (Fig. 4C).

**Figure 7:**
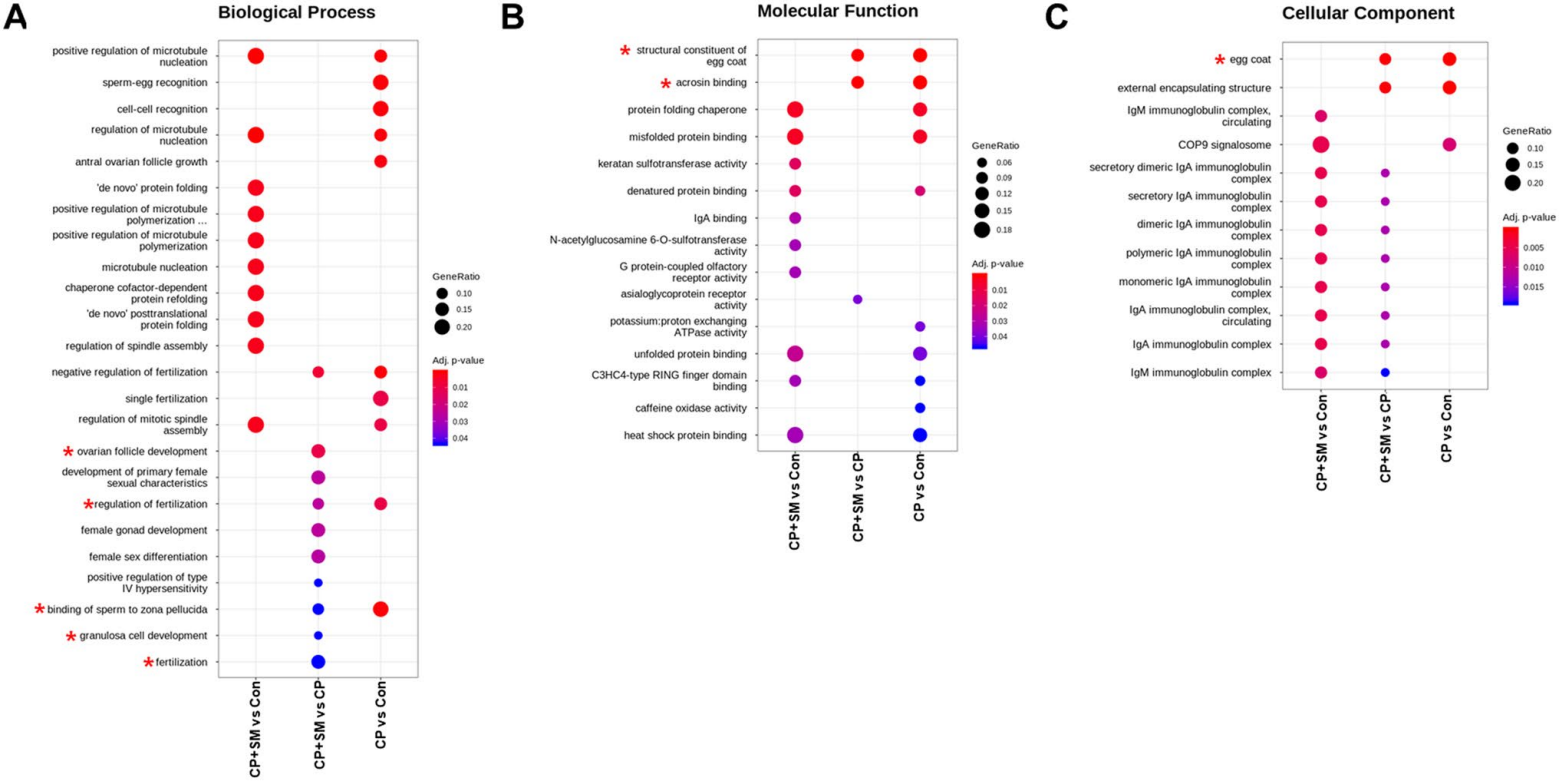
Gene Ontology (GO) and KEGG analysis of differentially expressed mRNAs. Targets were subjected to pathway analysis using KEGG and subsequently classified based on their enrichment in (**A**) biological process, (**B**) molecular function, and (**C**) cellular component GO terms. Asterisks (*) indicate the most important biological processes altered by SM administration compared to those in CP mice. *Con* saline-treated control mice; *CP* cyclophosphamide-treated mice; *CP + SM* CP-treated mice orally administered Samul-tang.

Gene ontology (GO) analysis of the CP and CP + SM mouse datasets revealed that the DEGs were enriched in biological processes such as ovarian follicle development and fertilisation (Fig. 7A), in molecular functions such as structural constituents of the egg coat and acrosin binding (Fig. 7B), and in cellular components such as the egg coat and secretory and circulating IgA and IgM immunoglobulin complexes (Fig. 7C).

### SM reversed CP-induced changes in the expression of microRNAs in mouse ovaries

A small number of microRNAs were differentially expressed in the three mouse groups. A comprehensive microarray tool showed the presence of 998.6 ± 37.4, 1,014.8 ± 26.8, and 1,052.2 ± 17.3 microRNAs in the Con, CP, and CP + SM mice, respectively (Supplementary Fig. S2A). Volcano plots showing differential microRNA expression among the three mouse groups are presented in Supplementary Fig. S2B.

**Figure 8:**
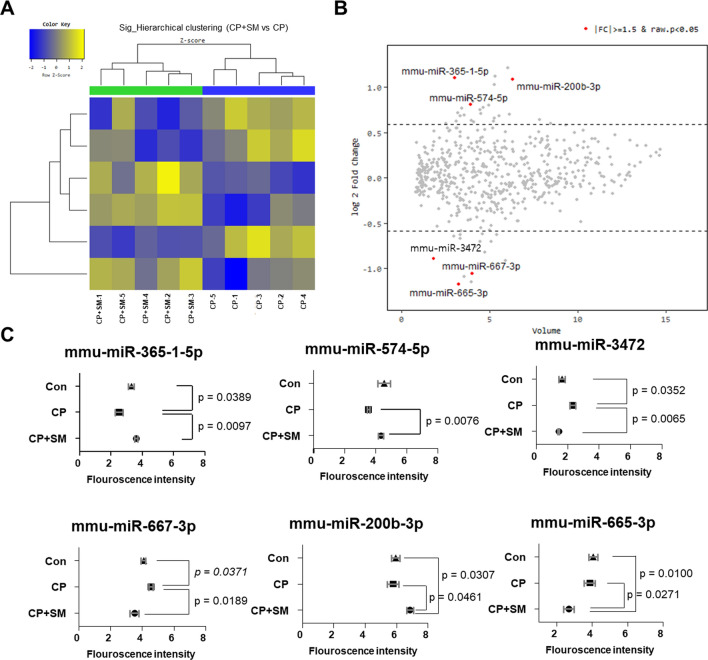
Hierarchical clustering and analysis of differentially expressed microRNAs. Microarray analysis was performed to compare the microRNA expression in ovulated ovaries of the CP and CP + SM mice. (**A**) Hierarchical clustering among the microRNA expression profiles. (**B**) Volume plot of log2 fold changes between the CP and CP + SM mice, showing six significantly differentially expressed microRNAs with fold changes > 1.5 and *P* < 0.05. (**C**) Microarray fluorescence intensities indicate the expression levels of miR-365-1-5p, miR-3472, miR-200b-3p, miR-665-3p, miR-667-3p, and miR-574-5p in the Con, CP, and CP + SM mice. *CP* cyclophosphamide-treated mice; *CP + SM* CP-treated mice orally administered Samul-tang.

Hierarchical clustering analysis and a volume plot revealed that the expression of six microRNAs was significantly different (fold-change > 1.5 and *P* < 0.05) between the CP + SM and CP mice (Fig. 8A,B). Interestingly, CP treatment decreased the expression of miR-365-1-5p and miR-574-5p and increased that of miR-3472 and miR-667-3p, whereas SM administration restored the expression levels of these microRNAs to those in the Con mice (Fig. 8C). Compared to the Con and CP mice, the CP + SM mice showed significant differential expression of two microRNAs, miR-200b-3p and miR-665-3p (Fig. 8C). Collectively, these findings indicate that SM administration may ameliorate the CP-induced impairment of ovarian function via epigenetic regulation.

### Integrative analysis of differentially expressed mRNAs and microRNAs

**Figure 9:**
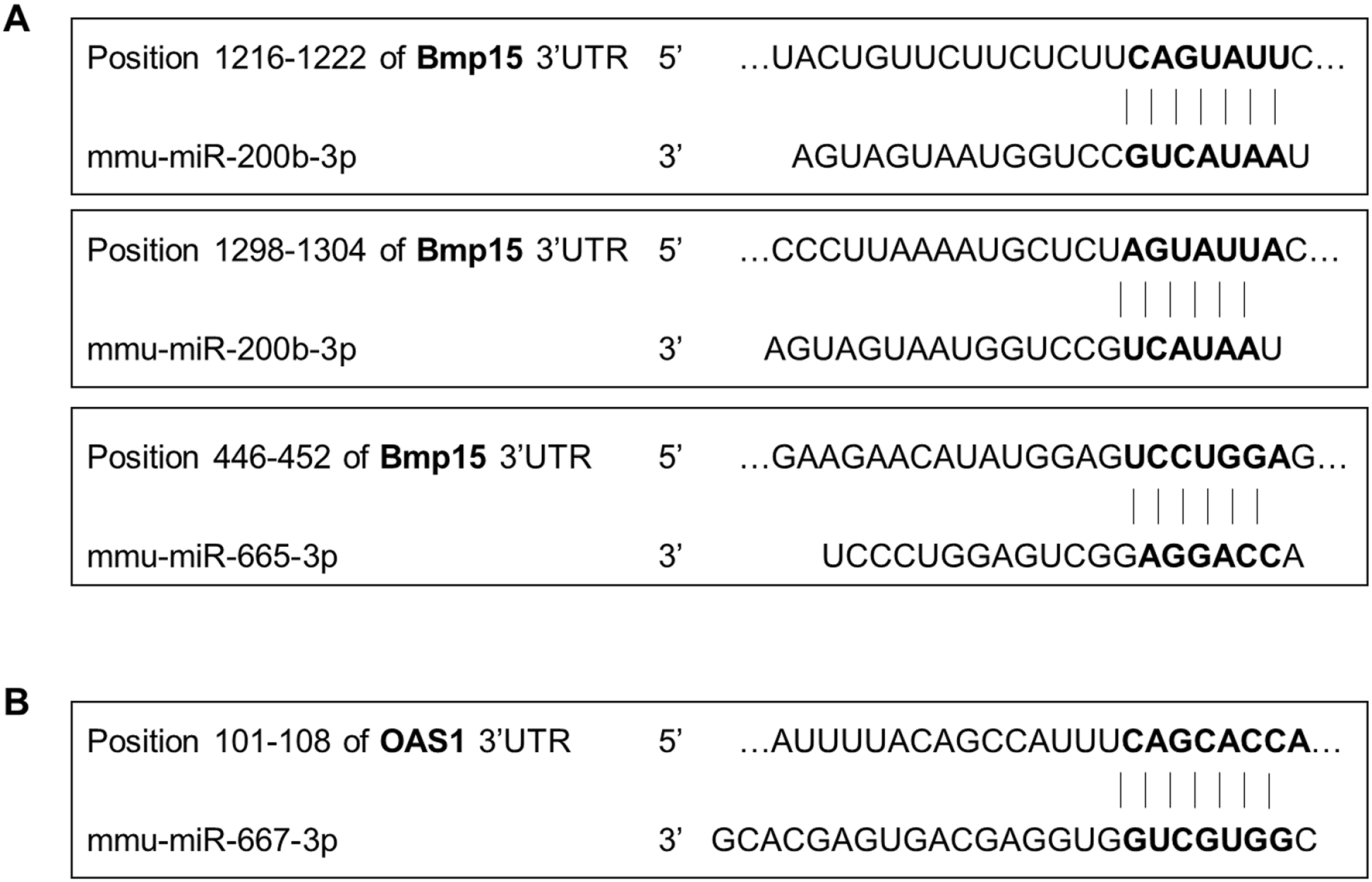
Binding sites between differentially expressed mRNAs and microRNAs. Binding sites in (**A**) *Bmp15* for miR-200b-3p and miR-665-3p and (**B**) *Oas1* for miR-667-3p, as predicted by TargetScan analysis.

Further enrichment analysis using the TargetScan webtool identified possible direct interactions between differentially expressed mRNAs and microRNAs (Fig. 9). We found that the *Bmp15* mRNA harboured binding sites for miR-200b-3p and miR-665-3p and that the *Oas1* family transcript harboured a binding site for miR-667-3p, of which expressions were changed by SM administration after CP exposure.

## Discussion

Traditional medicine-based treatment strategies for infertility vary between patients. SM has been traditionally used as an adjuvant herb in Korea to treat menstrual irregularities and infertility in women^18^. However, it remains unclear whether herbal treatments may improve clinical outcomes in patients with infertility. Therefore, in this study, we investigated the effects of SM as a fertoprotective adjuvant in a CP-induced DOR mouse model.

SM administration restored the LH/FSH ratio, which is known to be associated with follicular depletion^19,20^. These findings suggested that SM administration initiated the hormonal feedback regulatory system. Interestingly, the oocyte quality in mice significantly improved after SM administration without the protection of impaired physiological conditions and the loss of ovarian follicles. CP treatment is known to induce oxidative stress in granulosa cells and oocytes during folliculogenesis and ovulation. Furthermore, CP treatment induces ovulation of premature oocytes, thereby resulting in poor embryo quality^21^. SM has reportedly shown radical-scavenging activities against the hydroxyl radical and nitric oxide^22^, which are known to deteriorate the MII oocyte spindle and cause poor oocyte quality^23^. Our results demonstrated that SM exerted protective effects on oocytes with respect to chromosomal normality and spindle alignment, supposedly via its antioxidant activity. SM administration inhibited the CP-induced alterations in the spatiotemporal dynamics of oocyte maturation and aided in complete oocyte maturation.

To elucidate the underlying mechanism of the protective effects of SM, we performed microarray analysis to investigate the mRNA and microRNA expression in ovaries. SM administration reversed CP-induced alterations in the expression of genes related to the fertilisation potential, such as those involved in sperm-egg recognition, negative regulation of fertilisation, and regulation of spindle assembly. The DEGs identified between the CP and CP + SM groups were found to be involved in ovarian follicle development, binding of sperm to ZP, and microtubule nucleation. Of these, CP induced significant decreases in the expression of *Zp2*, *Zp3*, and *Nlrp5*, which was restored to normal levels after SM administration. Reduced mRNA levels of the *Zp2* and *Zp3* genes result in a low fertilisation rate and IVF failure in humans and mice^24,25^ Moreover, *Nlrp5* expression has been reported to decrease with maternal age, and *Nlrp5*-deficient human oocytes show mitochondrial depletion, resulting in the arrest of embryonic maturation^26,27^. However, the microarray and qPCR data of *Rhox8, Mterf1b,* and *Obox5* were in disagreement. These variations may be attributed to the filtering condition of microarray data for measures of quality including fold-change > 1.5 and *P* < 0.05 as previously described^28^. Further investigation is necessary for identifying the genetic regulation of SM. Importantly, the expression of oocyte-specific genes was restored in CP-treated mice after SM administration. BMP15, OAS1D, OBOX1, and OBOX5 are well-known oocyte-specific growth factors involved in ovarian folliculogenesis and oocyte maturation^29,30^, and downregulation of their encoding genes has been suggested to compromise fertility in women undergoing cancer treatment. *Bmp15* is known to regulate ovulation by preventing the overactivation of primordial follicles, and the expression of this gene is used as a marker of oocyte quality^31^. The reversal of the CP-induced decrease in the *Bmp15* expression by SM administration improved the quality of ovulated oocytes. Although little is known about the function of *Obox* family genes, their transcription is induced during zygotic gene activation, suggesting functional compensation between *Obox* family members^32,33^. Interestingly, among the differentially expressed microRNAs identified in our study, three microRNAs (miR-200b-3p, miR-665-3p, and miR-667-3p) have binding sites on either *Bmp15* or *Oas1* mRNAs. These results indicate that changes in the expression of microRNAs and their targets may be involved in the regulation of different fertility-related processes, from ovulation to fertilisation.

The expression of miR-365-1-5p, miR-3472, miR-574-5p, and miR-667-3p was restored in CP-treated mice at 4 weeks after SM administration. Little is known about the roles of miR-365-1-5p, miR-3472, and miR-667-3p. Meanwhile, one of the known functions of miR-574-5p is tumour suppression via the inhibition of MMP3 expression, which is important for tissue remodelling and extracellular matrix maintenance during the follicular and luteal phases^34^. Thus, SM-mediated restoration of the miR-574-5p expression may play a role in the protective effects of SM on folliculogenesis and ovulation in CP-treated mice. These changes in microRNA expression may be involved in the complex epigenetic regulation of ovarian function. Our study did not perform the functional analysis of differentially expressed mRNAs and microRNAs after SM administration in chronically impaired ovarian function. However, this is the first report on the effect of SM on CP-induced ovarian dysfunction using integrative microarray analysis. It provides scientific evidence on its use in adjuvant therapy.

The comprehensive microarray analysis performed in this study showed that the adverse effect of CP on ovarian function was sustained for 8 weeks after CP exposure, which is sufficient time to replace CP-damaged primordial and growing follicles surrounding oocytes. This time is considered equivalent to more than 6 months in humans^35,36^. Women subjected to anticancer therapy are advised to avoid conception for 6 months to up to 2 years to prevent adverse effects on the foetus^37^. For application in DOR patients after CP treatment, it should have a protective effect against chronic ovarian dysfunction.

Collectively, the differential mRNA and microRNA expression analysis and subsequent integrative analysis performed in this study highlight the protective effect of SM on ovarian function and oocyte maturation under chronically impaired conditions. This protective effect remained for at least 4 weeks after the termination of SM administration and led to an increase in the number of mature oocytes. Therefore, SM may be used to increase the oocyte yield in IVF patients with DOR after CP treatment, potentially improving the chances of IVF success.

## Methods

### Mice

All the experiments and analyses were conducted in accordance with the relevant guidelines and regulations. Experimental animal protocols were approved by the Institutional Animal Care and Use Committee at the Korea Institute of Oriental Medicine, Daejeon, Korea (approval number 19–019). Female 8-week-old C57BL/6 mice (Nara Biotech, Pyeongtaek, Korea) were housed under specific pathogen-free conditions. The mice were administered an intraperitoneal injection of saline (control or Con group) or an equal volume of 75 mg/kg CP (Sigma–Aldrich, St. Louis, MO, USA) at days 1, 3, and 5 for one week. To investigate the effect of SM, powdered SM (Hanpoong, Iksan, Korea) was dissolved in distilled water, and the CP-treated mice were orally administered either distilled water (CP group) or 5 g/kg SM (CP + SM group) five times a week for 4 weeks using feeding needles.

Mice from the three groups were separated within the same cage to synchronise their hormonal cycles^38^. All mice were sacrificed 4 weeks after the completion of SM administration. This post-treatment interval was selected because it provided sufficient time for the development of preantral follicles from the newly recruited primordial follicles^35^. Ovaries were removed and immediately placed into 4% paraformaldehyde (Biosesang, Seongnam, Korea) or liquid nitrogen for histological or microarray analysis, respectively.

### Enzyme-linked immunosorbent assay (ELISA) for hormonal assessment

At 4 weeks after SM administration, blood samples were collected from the mice, and sera were separated and stored at − 80 °C until analysis. The serum concentrations of FSH, LH, and AMH were measured using hormone-specific ELISA kits from Cusabio Biotech Co. (Wuhan, China), Enzo Life Sciences (Farmingdale, NY, USA), and MyBioSource (San Diego, CA, USA), respectively, according to standard protocols and the manufacturers’ instructions. For FSH, both intra- and inter-assay coefficients of variation (CVs) were < 15%, with a sensitivity of 2.5 mIU/mL. For LH, the intra- and inter-assay CVs were 7% and 15%, respectively, and the functional sensitivity was 5.2 mIU/mL. For AMH, the inter-assay CV was < 10%, with a sensitivity of 0.19 ng/mL.

### Histological assessment of ovarian follicles

At 4 weeks after SM administration, both ovaries were serially sectioned to obtain 5-μm-thick tissue sections, which were stained with haematoxylin and eosin. The cross-sectional equivalent diameters of ovaries were measured using NIS-Elements BR version 4.60.00 (Nikon Instruments, Tokyo, Japan) according to the manufacturer’s guidelines. Primordial, primary, secondary, and preovulatory follicles, with visible oocytes, were counted in every fifth stained section to avoid counting the same follicle twice. The follicular stages were classified as previously described^39^: primordial follicles, with a single flat layer of granulosa cells surrounding the oocyte; primary follicles, with a single cuboidal granulosa cell layer; secondary follicles, with at least two granulosa cell layers and a theca layer; and preovulatory follicles, with a complete antrum and theca layer.

### Assessment of oocyte quantity and quality

At 4 weeks after SM administration, the mice were superovulated via an intraperitoneal injection of 5 IU of pregnant mare serum gonadotropin (Prospec, Rehovot, Israel), followed by an injection of 5 IU of human chorionic gonadotropin (hCG; Prospec) 48 h later. Oocytes were collected at 18 h post-hCG injection into the preincubated human tubal fluid medium (Irvine Scientific, CA, USA). Oocytes were fixed with 4% paraformaldehyde (Biosesang) and permeabilised with 0.5% Triton X-100 (Sigma–Aldrich) for 10 min, followed by blocking with PBS containing 3% bovine serum albumin (GenDEPOT, TX, USA) and incubation with a rabbit anti-α-tubulin antibody (1:200; Cell Signaling Technology, MA, USA). Oocytes were mounted on slides using the VECTASHIELD antifade mounting medium with 4′,6-diamidino-2-phenylindole (Vector Laboratories, Peterborough, UK) to visualise the chromosomes and were observed under a fluorescent microscope (BX51; Olympus, Tokyo, Japan). Oocytes with well-organised bipolar spindles and tightly aligned chromosomes at the metaphase were scored as normal. The oocyte quality was evaluated by measuring morphometric parameters, including the areas of the whole oocyte, ooplasm, and PVS, using a computed image analysis system (Nikon Instruments).

### Microarray analysis of mRNA and microRNA expression

Four weeks after SM administration, the ovaries were collected from the mice post-ovulation, and total RNA was extracted using the RNeasy mini kit (Qiagen, Venlo, Netherlands) according to the manufacturer’s instructions. The purity and integrity of the extracted RNA were evaluated using a NanoDrop ND-1000 UV–Vis spectrophotometer (Thermo Fisher Scientific, Waltham, MA, USA). All the samples showed a high purity [optical density (OD)_260_/OD_280_ > 1.80] and integrity (RNA integrity number > 7.0). Hybridisation with GeneChip Mouse Gene 2.0 ST arrays (Affymetrix) was controlled using the GeneChip Command Console software. MicroRNA expression was analysed using the Affymetrix GeneChip microRNA array 4.0, based on MirBase release 20. The Affymetrix Expression Console 1.4 software was used for basic data extraction (CEL files) and quality control metrics. A fold-change value > 1.5 and a *P-*value < 0.05 were used as thresholds to identify DEGs.

### Integrative analysis of mRNA and microRNA expression profiles

Gene enrichment and functional annotation analysis for a significant probe list were performed using GO (http://geneontology.org) and the Kyoto Encyclopedia of Genes and Genomes (KEGG; http://kegg.jp) to identify the potential functions of DEGs in the biological process, molecular function, and cellular component categories. Data analyses and visualisation of DEGs were performed using R 3.5.1 (www.r-project.org). An integrative analysis was performed using the TargetScan webtool 7.2 (www.targetscan.org, accessed March 2018) to find binding sites for critical microRNAs and DEGs.

### Validation of selected differentially expressed gene expression in the ovaries

To confirm mRNA microarray results, validation was performed on significant genes of interest using real-time quantitative PCR (qPCR). The synthesis of complementary DNAs from extracted total RNA was performed using iScript cDNA Synthesis kit (Bio-Rad Laboratories, Hercules, CA) according to the manufacturer’s instructions. The qPCR was performed in a final reaction volume of 20 µL using QuantStudio 6 Flex Real-time PCR System with fluorescent probe-based Taqman assays according to the manufacturer’s instructions (Thermo Fisher Scientific). The cycle threshold was normalised and compared using *Gapdh* as the internal standards.

### Statistical analysis

The statistical significance of differences between two groups was determined by the Student’s *t*-test using GraphPad Prism version 8.4.0 (GraphPad Software, Inc., La Jolla, CA, USA). Differences with *P* < 0.05 were considered statistically significant.

## Supplementary Information

Supplementary Information.

## Data Availability

The datasets generated and/or analysed during the current study are available from the corresponding author on reasonable request.
