# Supplementary material for: Samul-tang ameliorates oocyte damage due to cyclophosphamide-induced chronic ovarian dysfunction in mice
Source: Sci Rep. 2020 Dec 14;10:21925. doi: 10.1038/s41598-020-79013-7 (PMC7736329; doi:10.1038/s41598-020-79013-7)
Supplement: Supplementary file 1 — Supplementary Information. [file 41598_2020_79013_MOESM1_ESM.docx]

**Supplementary material**

**Samul-tang ameliorates oocyte damage due to cyclophosphamide-induced chronic ovarian dysfunction in mice**

Jihyun Kim, Sooseong You*


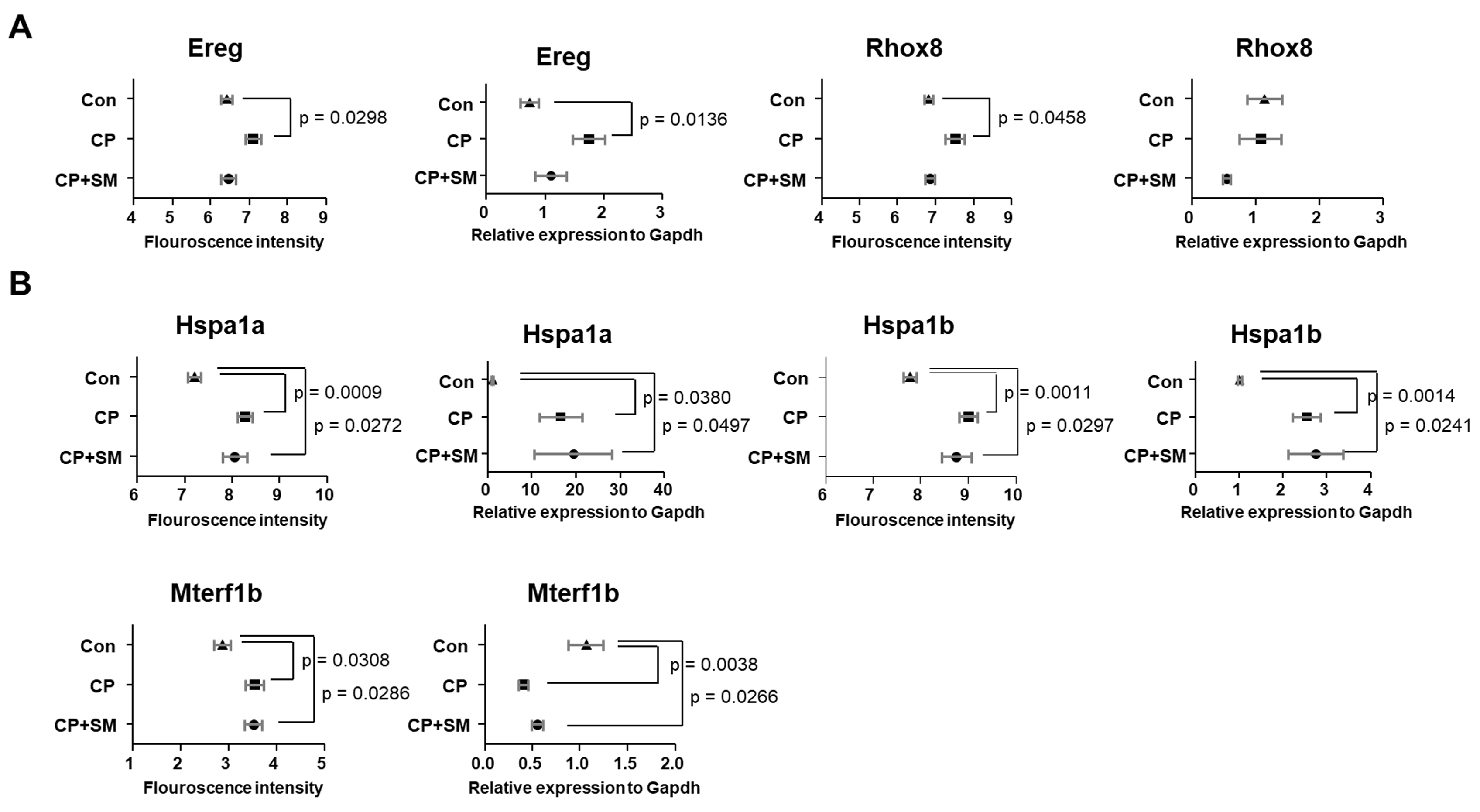


**Supplementary Figure 1. Differentially expressed mRNAs related to ovarian function in mouse ovaries.** Microarray fluorescence intensities and Taqman assay-based validation of the expression of (A) *Ereg* and *Rhox8*, involved in folliculogenesis and oocyte ovulation; and (B) *Hspa1a*, *Hspa1b*, and *Mterf1b*, involved in mitotic spindle assembly and protein folding in Con, CP, and CP+SM mice. Statistical analysis was performed using the Student’s *t*-test. Con: saline-treated control mice; CP: cyclophosphamide-treated mice; CP+SM: CP-treated mice orally administered Samul-tang.

**Supplementary Figure 2. Comparison of microRNA profiles in the ovaries of Con, CP, and CP+SM mice.** (A) Numbers of microRNAs detected in the three mouse groups. (B) Volcano plot showing differential expression among the Con, CP, and CP+SM mice. Blue and yellow dots indicate significantly downregulated and significantly upregulated genes, respectively, with fold changes > 1.5 and *P* < 0.05. Con: saline-treated control mice; CP: cyclophosphamide-treated mice; CP+SM: CP-treated mice orally administered Samul-tang.


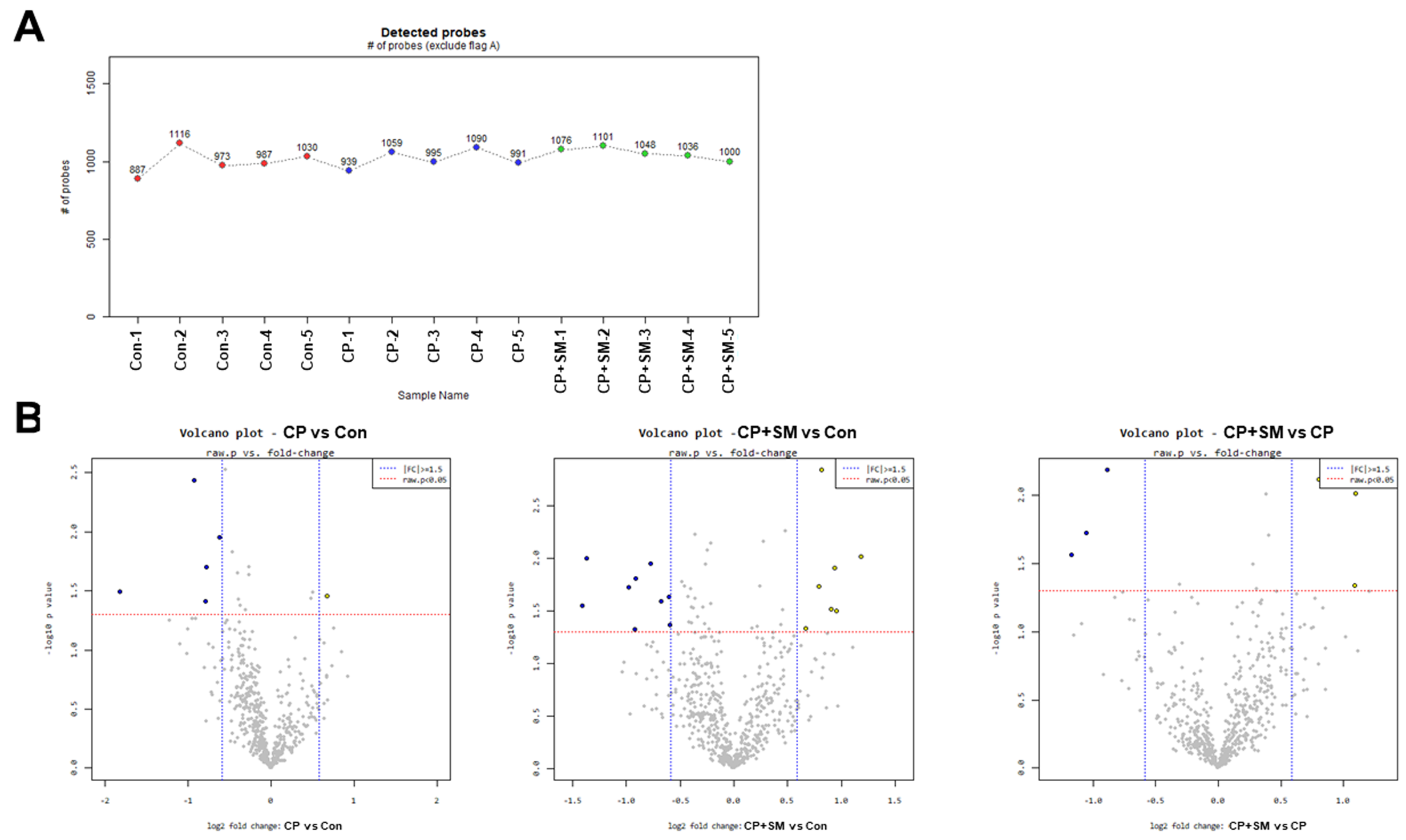


**Supplementary Table 1. List of genes upregulated in CP+SM mice compared to their expression in CP mice.**

| \| **Gene_Symbol** \| **Gene_ID** \| **CP+SM vs CP.fc** \| **P-value** \| \| --- \| --- \| --- \| --- \| \| Igkv6-23 \| immunoglobulin kappa variable 6-23 \| 2.063750 \| 0.00256 \| \| Ighv1-34 \| immunoglobulin heavy variable 1-34 \| 1.880408 \| 0.00891 \| \| Gm10944 \| predicted gene 10944 \| 1.847761 \| 0.03551 \| \| Gm22805 \| predicted gene, 22805 \| 1.756152 \| 0.01467 \| \| Nlrp5 \| NLR family, pyrin domain containing 5 \| 1.741490 \| 0.01785 \| \| Jchain \| immunoglobulin joining chain \| 1.741258 \| 0.02246 \| \| Oas1d \| 2-5 oligoadenylate synthetase 1D \| 1.733841 \| 0.01224 \| \| Nlrp14 \| NLR family, pyrin domain containing 14 \| 1.726038 \| 0.01331 \| \| Ighv7-3 \| immunoglobulin heavy variable 7-3 \| 1.691466 \| 0.0113 \| \| Zp2 \| zona pellucida glycoprotein 2 \| 1.675853 \| 0.01289 \| \| Padi6 \| peptidyl arginine deiminase, type VI \| 1.650908 \| 0.00743 \| \| Gm15698 \| transcription elongation factor B (SIII), polypeptide 2 pseudogene \| 1.648685 \| 0.00937 \| \| Ighv9-3 \| immunoglobulin heavy variable V9-3 \| 1.632920 \| 0.02819 \| \| Zp3 \| zona pellucida glycoprotein 3 \| 1.629114 \| 0.00616 \| \| Gm1965 \| predicted gene 1965 \| 1.608484 \| 0.007 \| \| Mir337 \| microRNA 337 \| 1.591651 \| 0.02867 \| \| Iglv2 \| immunoglobulin lambda variable 2 \| 1.590376 \| 0.04011 \| \| Mir467f \| microRNA 467f \| 1.587130 \| 0.01719 \| \| Nlrp9b \| NLR family, pyrin domain containing 9B \| 1.560798 \| 0.01238 \| \| Bmp15 \| bone morphogenetic protein 15 \| 1.553110 \| 0.00981 \| \| Gm24762 \| predicted gene, 24762 \| 1.553084 \| 0.02296 \| \| Slc18a2 \| solute carrier family 18 (vesicular monoamine), member 2 \| 1.552010 \| 0.01389 \| \| Gm24149 \| predicted gene, 24149 \| 1.543638 \| 0.02598 \| \| 2210409E12Rik \| transcription elongation factor B (SIII), polypeptide 2 pseudogene \| 1.531299 \| 0.01582 \| \| Mir1960 \| microRNA 1960 \| 1.529290 \| 0.02813 \| \| C86187 \| expressed sequence C86187 \| 1.528637 \| 0.02172 \| \| Gm25813 \| predicted gene, 25813 \| 1.518943 \| 0.0092 \| \| C87414 \| expressed sequence C87414 \| 1.517613 \| 0.02811 \| \| Igkj1 \| immunoglobulin kappa joining 1 \| 1.510140 \| 0.04913 \| \| Obox1 \| oocyte specific homeobox 1 \| 1.509595 \| 0.02126 \| \| Ighv14-4 \| immunoglobulin heavy variable 14-4 \| 1.503570 \| 0.01659 \| |
| --- | --- | --- | --- | --- | --- | --- | --- | --- | --- | --- | --- | --- | --- | --- | --- | --- | --- | --- | --- | --- | --- | --- | --- | --- | --- | --- | --- | --- | --- | --- | --- | --- | --- | --- | --- | --- | --- | --- | --- | --- | --- | --- | --- | --- | --- | --- | --- | --- | --- | --- | --- | --- | --- | --- | --- | --- | --- | --- | --- | --- | --- | --- | --- | --- | --- | --- | --- | --- | --- | --- | --- | --- | --- | --- | --- | --- | --- | --- | --- | --- | --- | --- | --- | --- | --- | --- | --- | --- | --- | --- | --- | --- | --- | --- | --- | --- | --- | --- | --- | --- | --- | --- | --- | --- | --- | --- | --- | --- | --- | --- | --- | --- | --- | --- | --- | --- | --- | --- | --- | --- | --- | --- | --- | --- | --- | --- | --- | --- |

Comparison of the CP and CP+SM mouse data revealed 31 differentially upregulated genes with fold changes > 1.5 and *P* < 0.05. CP: cyclophosphamide-treated mice; CP+SM: CP-treated mice orally administered Samul-tang.

**Supplementary Table 2. List of genes downregulated in CP+SM mice compared to their expression in CP mice.**

| **Gene_Symbol** | **Gene_ID** | **CP+SM vs CP.fc** | **P-value** |
| --- | --- | --- | --- |
| Gm16381 | predicted gene 16381 | 1.501364 | 0.04261 |
| Car12 | carbonic anyhydrase 12 | 1.509805 | 0.00919 |
| Gm25528 | predicted gene, 25528 | 1.545629 | 0.01206 |
| Taar7d | trace amine-associated receptor 7D | 1.557496 | 0.0277 |
| Trbj1-7 | T cell receptor beta joining 1-7 | 1.565902 | 0.02713 |
| Fmo2 | flavin containing monooxygenase 2 | 1.568672 | 0.0206 |
| Gm25162 | predicted gene, 25162 | 1.570509 | 0.00927 |
| Mir3473b | microRNA 3473b | 1.576593 | 0.02394 |
| Obox5 | oocyte specific homeobox 5 | 1.677008 | 0.00132 |
| Asgr1 | asialoglycoprotein receptor 1 | 1.761382 | 0.00261 |
| Gm22938 | predicted gene, 22938 | 1.792484 | 0.00031 |
| A730049H05Rik | RIKEN cDNA A730049H05 gene | 1.793160 | 0.03026 |
| n-R5s60 | nuclear encoded rRNA 5S 60 | 1.901513 | 0.00733 |

Comparison of the CP and CP+SM mouse data revealed 13 differentially downregulated genes with fold changes > 1.5 and *P* < 0.05. CP: cyclophosphamide-treated mice; CP+SM: CP-treated mice orally administered Samul-tang.
